# Polymicrobial airway bacterial communities in adult bronchiectasis patients

**DOI:** 10.1186/1471-2180-14-130

**Published:** 2014-05-20

**Authors:** Paul Purcell, Hannah Jary, Audrey Perry, John D Perry, Christopher J Stewart, Andrew Nelson, Clare Lanyon, Darren L Smith, Stephen P Cummings, Anthony De Soyza

**Affiliations:** 1 Department of Applied Sciences, Ellison Building, University of Northumbria, Newcastle upon Tyne NE1 8ST, England; 2 Transplantation and Immunobiology Group, Institute of Cellular Medicine, Newcastle University NE2 4HH and Adult Bronchiectasis Unit Freeman Hospital, Newcastle upon Tyne NE7 7DN, England; 3 Department of Microbiology, Freeman Hospital, Newcastle upon Tyne, UK; 4 Adult Cystic Fibrosis Unit, Department of Respiratory Medicine, Royal Victoria Hospital, Newcastle upon Tyne NE7 7DN, England

**Keywords:** Bronchiectasis, Bacterial colonisation, Sputum

## Abstract

**Background:**

Chronic airway infection contributes to the underlying pathogenesis of non-cystic fibrosis bronchiectasis (NCFBr). In contrast to other chronic airway infections, associated with COPD and CF bronchiectasis, where polymicrobial communities have been implicated in lung damage due to the vicious circle of recurrent bacterial infections and inflammation, there is sparse information on the composition of bacterial communities in NCFBr. Seventy consecutive patients were recruited from an outpatient adult NCFBr clinic. Bacterial communities in sputum samples were analysed by culture and pyrosequencing approaches. Bacterial sequences were analysed using partial least square discrimination analyses to investigate trends in community composition and identify those taxa that contribute most to community variation.

**Results:**

The lower airway in NCFBr is dominated by three bacterial taxa *Pasteurellaceae*, *Streptococcaceae* and *Pseudomonadaceae*. Moreover, the bacterial community is much more diverse than indicated by culture and contains significant numbers of other genera including anaerobic *Prevotellaceae*, *Veillonellaceae* and *Actinomycetaceae*. We found particular taxa are correlated with different clinical states, 27 taxa were associated with acute exacerbations, whereas 11 taxa correlated with stable clinical states. We were unable to demonstrate a significant effect of antibiotic therapy, gender, or lung function on the diversity of the bacterial community. However, presence of clinically significant culturable taxa; particularly *Pseudomonas aeruginosa* and *Haemophilus influenzae* correlated with a significant change in the diversity of the bacterial community in the lung.

**Conclusions:**

We have demonstrated that acute exacerbations, the frequency of exacerbation and episodes of clinical stability are correlated, in some patients, with a significantly different bacterial community structure, that are associated with a presence of particular taxa in the NCFBr lung. Moreover, there appears to be an inverse relationship between the abundance of *P. aeruginosa* and that of of *H. influenzae* within the NCFBr lung bacterial community. This interaction requires further exploration.

## Background

Bronchiectasis is a significant cause of chronic respiratory disease resulting in irreversible abnormally dilated bronchi associated with chronic inflammation, chronic cough and sputum production [1]. It can be caused by physical obstruction or post infectious damage, genetic defects (as observed in cystic fibrosis), abnormal host defence or autoimmune disease but in many cases bronchiectasis is idiopathic [2]. In this study we have focussed on the examination of a cohort of patients that presented with non-CF bronchiectasis (NCFBr).

Chronic airway infection contributes to the underlying pathogenesis of the disease, with progressive lung damage resulting from recurrent bacterial infections and inflammatory responses [3]. The most commonly cultured pathogens associated with sputum of NCFBr are *Haemophilus influenzae* and *Pseudomonas aeruginosa* with many isolated strains showing significant antibiotic resistance [1,4]. In prior studies, individuals that were culture-negative for bacterial pathogens showed the mildest disease, whereas, those with *P. aeruginosa* had the most severe bronchiectasis [5]. Consequently, the presence and persistence of *P. aeruginosa* has been identified as a marker of bronchiectasis severity, although it remains unclear whether this is causal or associated with accelerated lung function decline [6].

Frequent exacerbations experienced by bronchiectasis patients may contribute to the progressive decline of lung function [7], though both the aetiology and pathophysiology of exacerbations remains poorly understood. Exacerbations are frequently managed with antibiotics, however, viral infections may also be significant in many cases but their role requires clarification [1].

The aim of this study was to investigate the airway microbiota in NCFBr and characterise its diversity and structure. We aimed to test the hypotheses that bacterial community differences reflect the exacerbation history of the patient, that the presence or absence of culturable pathogens sculpted the structure of the airway microbiome and that the bacterial community would show significant change in response to the interventions used to manage patient outcomes.

## Results

### Patient cohort

Patient baseline data are summarised in Table 1. The study population consisted of 25 males and 45 females. The self-reported exacerbation rates in the preceding 12 months were available for 61 of the 70 patients. Thirty-eight patients were identified as frequent exacerbators with more than 3 exacerbations in a 12 month period**.** At the time of sample collection 20 patients reported symptoms consistent with exacerbation (Additional file 1: Table S1).

**Table 1:** Patient data for the cohort

**Demographic data**	**All patients (n = 70)**	**Non-exacerbated (n = 50)**	**Exacerbated (n = 20)**
Age (yr)	61.6 ± 13	61.2 ± 13.4	62.5 ± 13
Female (%)	64.3	60	75
FEV (L)	1.46	1.45	1.54
Males	1.78a	1.80a	1.77a
Females	1.26b	1.20b	1.45b
FEV_1_% predicted	57.9	55.2	64.9
Frequent exacerbation (%)^*^ (n = 61)	61.7	56	45
Culture negative (%)	38.6	22	40
*H. influenzae* colonisation (%)	21.4	12	45
*P. aeruginosa* colonisation (%)	32.8	40	15
Recent Antibiotics (%)^+^	24.3	22	30

*Frequency of exacerbation data (available for 61 patients). Frequent exacerbators defined as >3 episodes per annum. + Indicates treatment within the last month with antibiotics other than maintenance colomycin or azithromycin. Values followed by different letters are significantly different (p < 0.05). When corrected for sex and height the FEV1% predicted were similar between the 2 genders.

### Microbial culture

Sputa from 51 patients (73%) were culture positive for pathogenic microorganisms, the remainder either yielded no bacteria or non-pathogenic mixed oral flora as determined by the standard culture protocol used in the clinic (Additional file 1: Table S1). The most common organisms were *P. aeruginosa* found in 33% and *H. influenzae* in 21% of patients respectively. There were no instances of both *P. aeruginosa* and *H. influenzae* being found within a single sputum sample. Patient records showed that 24 individuals had *P. aeruginosa* isolated from all previous sputum samples referred for culture; these patients were regarded as persistently colonised. There were 17 patients regarded as intermittently colonised, with *P. aeruginosa* isolated from at least one but not all sputa samples and 29 patients were culture negative. The majority (71%) of frequent exacerbators (n = 38) were culture positive for lung pathogens. Of these individuals, 50% were colonised with *P. aeruginosa* and 10.5% with *H. influenzae*.

### The relationship between culture status and lung function

Lung function, was determined by forced expiratory volume in one second (FEV1% predicted). In patients harbouring *H. influenzae* or where culturable pathogens were absent FEV1% predicted was 64.5 and 64.9 respectively, these values were significantly higher (*P =* 0.0002 and *P =* 0.0001 respectively) in comparison with individuals whose sputum was culture positive for *P. aeruginosa* (FEV1% predicted = 48.5). Lung function was significantly lower (P < 0.001) in patients persistently colonised with *P. aeruginosa* (FEV1% predicted = 40.6) compared those ‘never’ or intermittently colonised by this pathogen (FEV1% predicted 59.7 and 69.8 respectively). In contrast, those never colonised and those intermittently colonised did not have significantly different FEV1% predicted values. Patients who frequently exacerbated (FEV1% predicted = 58.8) and those that did not (FEV1% predicted = 59.3) had no significant difference in lung function.

### The bacterial community structure derived by 16S rRNA gene amplicon pyrosequencing

Pyrosequencing data (Additional file 2: Figure S1) revealed that the sputum samples contained on average 50 individual families (range 13–144). Bacterial community diversity was not significantly different between genders. Community diversity was not significantly correlated with FEV1% predicted (*P =* 0.28).

There were three dominant families in the sputa, the first was *Pseudomonadaceae*, where a single operational taxonomic unit (OTU) contributed 92% of all the reads for this taxa. Comparison with culture data and analyses of the sequence data to putative species level (Additional file 3: Table S2) indicated this OTU was *P. aeruginosa.* The second major taxa was *Pasteurellaceae*, 84% of reads for this family belonged to a single OTU that culture data and sequence analyses to putative species level indicated was *H. influenzae*. A further 9% of the remaining reads belonged to a second OTU, found in only one patient (BX16), from which only *H. parainfluenzae* had been cultured. The third abundant taxa belonged to *Streptococcaceae*, where two OTUs contributed 88% of all reads for this group.

Culture analyses of the sputum samples (Table 1) indicated that 27% of the patients were negative for organisms regarded as of concern clinically. However, sequence data showed that these individuals had significantly greater numbers of taxa present than culture-positive patients (average 63 versus 46 taxa, P = 0.011). The predominant organisms in culture negative samples were *Streptococcaceae*, representing on average 48% of the total community in these samples which are likely to represent those streptococci that are not detected by the standard culture techniques used to analyse the sputum. *Pseudomonadaceae* was found to contribute to the community composition, although not detectable by culture, however, in exacerbating samples it contributed, on average 0.2% whereas, in stable samples it contributed 13% to the total community composition.

### The role of environmental variables in driving bacterial community structure

**Figure 1:**
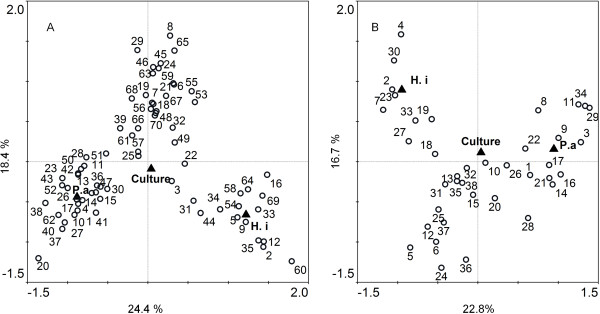
**Canonical correspondence analysis of (A) total cohort showing that sputum samples that were culture positive (*P =*** **0.016); had culturable *P. aeruginosa *(*P =*** **0.002) and culturable *H. influenzae *(*P =*** **0.002) were associated with distinct bacterial community assemblies, (B) Frequent exacerbators also showing that samples that were culture positive (*P =*** **0.05); had culturable *P. aeruginosa *(*P =*** **0.002) and culturable *H. influenzae *(*P =*** **0.002) were associated with distinct bacterial community assemblies.** Discrete variables, indicated by ▲, are; Culture positive sputum; H.i, *H. influenzae* detected by culture and P.a, *P. aeruginosa* detected by culture. Other variables analysed; the presence of an exacerbation at time of sampling; 12 month history of persistent; intermittent or absence of culturable *P. aeruginosa*, current azithromycin treatment; current nebulised colistin treatment; gender, FEV1% predicted; frequent exacerbation and age. None were found to significantly affect the community structure and for clarity are not shown. Percentage values show variance within data explained by that axis.

In order to investigate impact of patient specific and clinical variable on the bacterial community structures the bacterial profiles from the patient cohort were subjected to ordination analysis (CCA) and permutation testing. Analyses were performed on the bacterial community resolved to family level then repeated with putative species level resolution. These analyses constrained the community profile variance with 13 measured variables (Culture positive sputum; *H. influenzae* detected by culture; *P. aeruginosa* detected by culture; the presence of an exacerbation at time of sampling; 12 month history of persistent; intermittent or absence of culturable *P. aeruginosa*, antibiotic treatment in previous month; current azithromycin treatment; current nebulised colistin treatment; gender, FEV1% predicted and age) derived from patient records. For the family level analyses, these variables explained 64.4% of the total variance in the data but only three were significantly associated with the variance in bacterial community structure. These were culture positive sputum (*P =* 0.016); the presence of culturable *H. influenzae* (*P =* 0.002); and culturable *P. aeruginosa* (*P =* 0.002) (Figure 1A).

These analyses were repeated for the frequent exacerbator cohort all of whom had received antibiotic intervention in the preceding 12 months. These variables contributed to 62% of the variance in the community structure but significant associations between the microbial community structures were limited to culture-positive sputum (*P =* 0.05), the isolation of *H. influenzae* (*P =* 0.002) and the isolation of *P. aeruginosa* (*P =* 0.002) (Figure 1B).

Repeating these analyses at putative species level resolution found the same result, with only these three variables showing significant associations with the bacterial community structure.

The presence of culturable *H. influenzae* and culturable *P. aeruginosa* exerting significant effects on community structure was supported by examination of the read numbers of these taxa in the pyrosequencing analysis. When one species was present (with one exception, patient 63), then the other species did not contribute more than 1.5% to the total bacterial community profile (Additional file 2: Figure S1).

The other variables analysed were the presence of an exacerbation at time of sampling; 12 month history of persistent; intermittent or absence of culturable *P. aeruginosa*; current azithromycin treatment; current nebulised colistin treatment; gender, FEV1% predicted; antibiotic treatment in previous month and age. None were found to significantly affect the community structure in either the total or frequently exacerbating cohorts. Of particular interest were 25 patients that had not received antibiotics for one month prior to sample collection. Ordination analyses (Figure 1A) showed that these individuals did not have significantly different bacterial communities to those who were receiving antibiotic therapy.

### Bacterial community structure and clinical status

**Figure 2:**
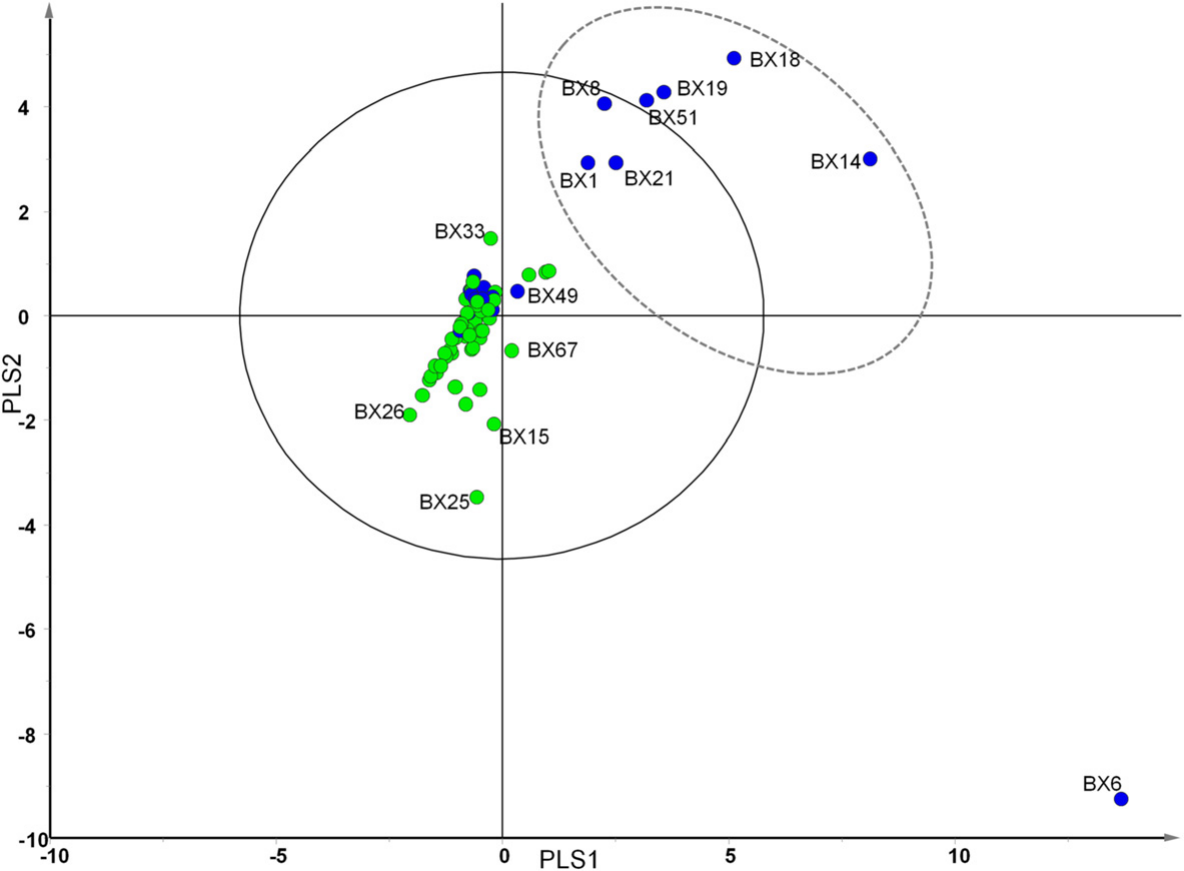
**Partial least squares discriminant analysis (PLS-DA) loading plot based on the relative abundance of bacterial taxa determined by 454 sequence analysis of the microbiota of sputum from patients reporting current stability (green circle) and sputum from patients reporting a current exacerbation (blue circle).** PLS1 (*R*^*2*^*X* = 0.169, *R*^*2*^*Y* = 0.232, *Q*^*2*^ = 0.0287) and PLS 2 (*R*^*2*^*X* = 0.107, *R*^*2*^*Y* = 0.124, *Q*^*2*^ = 0.0601) are given. The solid ellipse indicates Hotellings *T*^*2*^ range, at 95% confidence. Patient samples derived from current exacerbators contained within the dashed ellipse, and including BX6 are deemed to be the major outliers, having a microbial community composition which is dissimilar to the stable and a small proportion of exacerbating patients. Some sample labels have been removed for ease of interpretation.

For partial least squares discriminant analysis (PLS-DA), samples were classified according to exacerbation status with group 1 (n = 50) being stable and group 2 (n = 20) exacerbating at time of sampling. The model made no further assumptions about each patient group. Analysis of the scatter plot of scores (Figure 2), demonstrated that 8 individuals from the exacerbating group (40%) had bacterial community structures that were distinct from those of the remaining patients. Within the 20 individuals sampled during an exacerbation, 12 patients exhibited a community composition that was similar to 22 patients who were stable at time of sampling in terms of projection in the XY space. The remaining 28 stable patients had a community composition that was distinct from the remaining 8 exacerbation patients (Figure 2).

**Figure 3:**
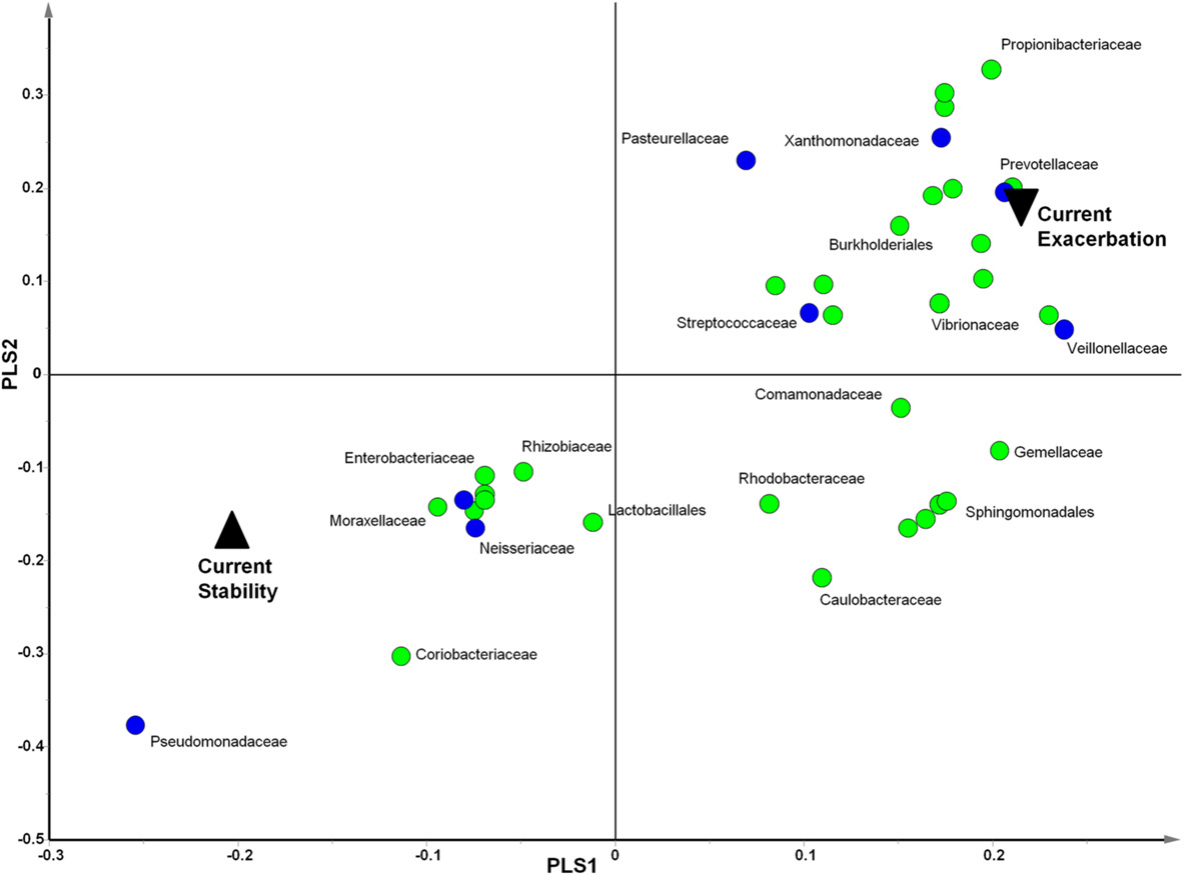
**Partial least squares discriminant analysis (PLS-DA) loading plot showing the contributing microbial community members towards the separation of the PLS-DA scores between patients reporting current stability (▲) and sputum from patients reporting a current exacerbation (▼).** PLS1 (R2X = 0.169, R2Y = 0.232, Q2 = 0.0287) and PLS 2 (R2X = 0.107, R2Y = 0.124, Q2 = 0.0601) are given. Taxa deemed clinically relevant (based on those screened during standard culture) are highlighted in blue. Some sample labels have been removed for ease of interpretation.

Eleven bacterial taxa, including members of *Pseudomonas*, *Neisseria* and Enterobacteriales were associated with the stable clinical state. Conversely, 27 taxa were positively correlated with exacerbation, including Burkholderiales, *Pasteurellaceae*, *Streptococcaceae*, *Xanthomonadaceae*, *Prevotellaceae* and *Veillonellaceae* as well as other taxa not regarded as pathogens (*Propionibacterium*, Flavobacteriales and Actinobacteria) (Figure 3).

### Bacterial community analysis of the lung microbiota from frequently exacerbating patients

**Figure 4:**
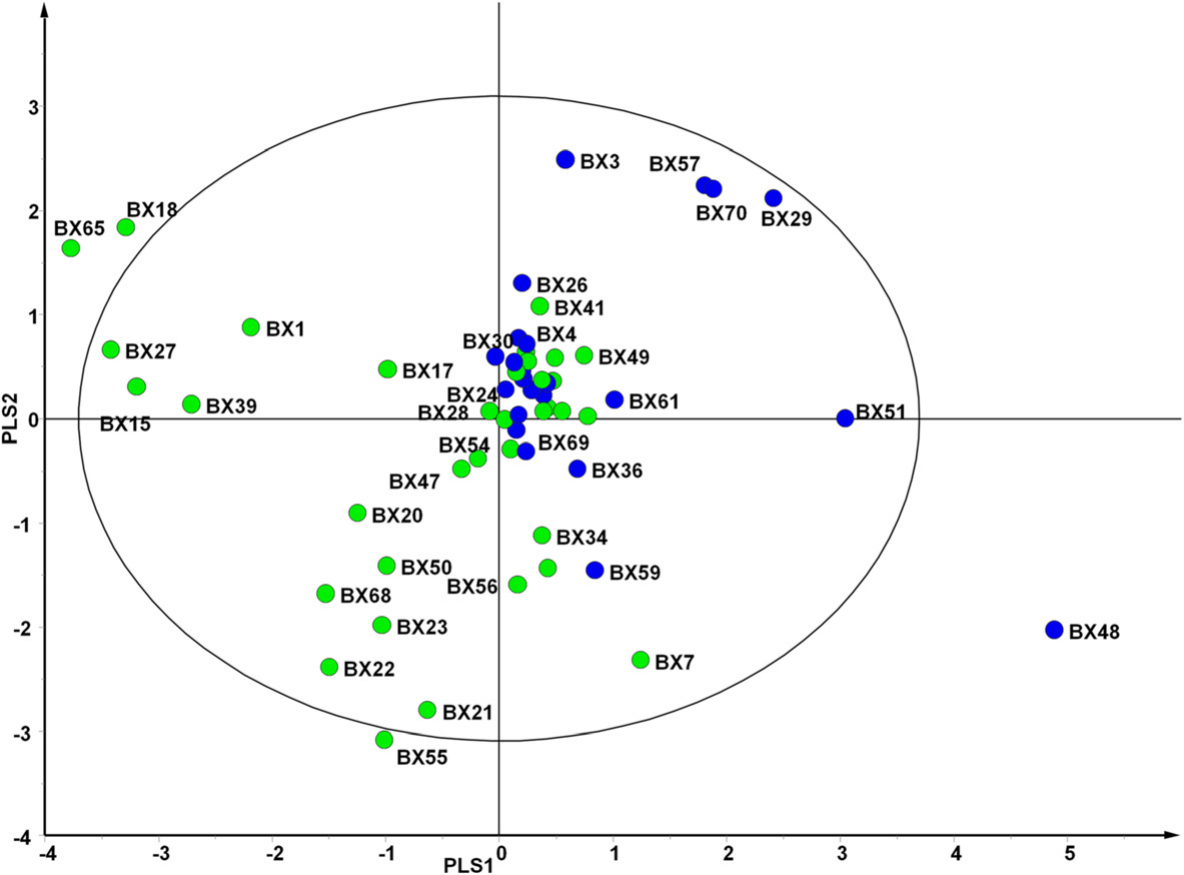
**Partial least squares discriminant analysis (PLS-DA) loading plot based on the relative abundance of bacterial taxa determined by 454 sequence analysis of the microbiota of sputum from patients with greater than 3 exacerbation event per annum (green circle) and sputum from patients with 3 or fewer exacerbation events per annum (blue circle).** PLS1 (*R*^*2*^*X* = 0.0701, *R*^*2*^*Y* = 0.232, *Q*^*2*^ = 0.0467) and PLS 2 (*R*^*2*^*X* = 0.0477, *R*^*2*^*Y* = 0.124, *Q*^*2*^ = 0.0601) are given. The solid ellipse indicates Hotellings *T*^*2*^ range, at 95% confidence. Patient samples falling outside of the ellipse are deemed to be the major outliers. Some sample labels have been removed for ease of interpretation.

**Figure 5:**
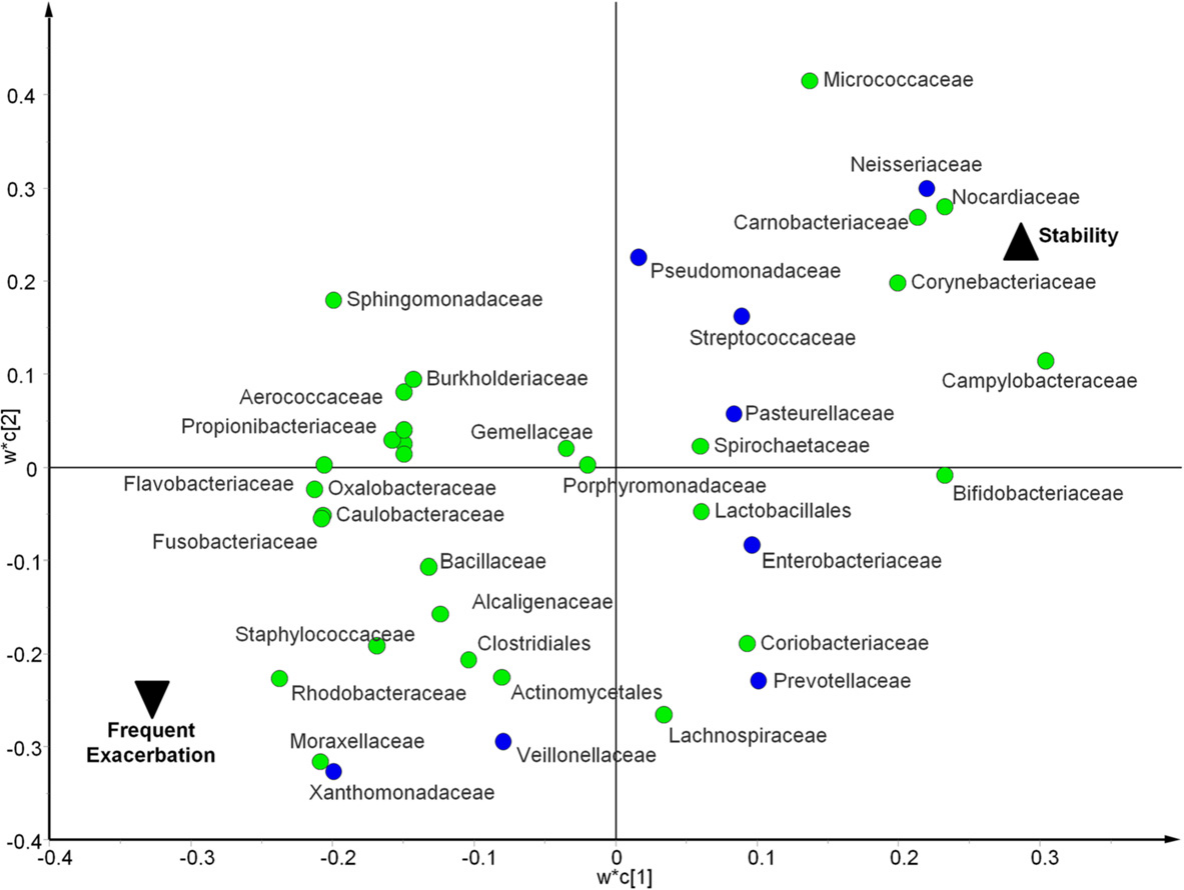
**Partial least squares discriminant analysis (PLS-DA) loading plot showing the contributing microbial community members towards the separation of the PLS-DA scores between patients are frequent exacerbators (>3 exacerbation events per annum) and sputum from patients who are stable (≤3 exacerbation events per annum).** Taxa deemed clinically relevant (based on those screened during standard culture) are highlighted in blue. Some sample labels have been removed for ease of interpretation.

Analytical models were extended to explore any differences in prior exacerbation history. From the cohort, 59 patients were selected for inclusion in the model. Patients were defined as frequently exacerbating (M1, n = 38 having more than 3 exacerbation events per annum) or stable (M2, n = 23, ≤3 event pa). Analysis of the model showed that 22 patients from M1 and 17 from M2 had bacterial profiles that were similar, despite exacerbation history (indicated with an ellipse, Figure 4). The remaining 20 patient samples, however, could be stratified between stable and frequent exacerbation states (Figure 4). Further analysis of the overall bacterial community structure between frequent exacerbating (M1) and stable (M2) patients revealed *Moraxellaceae*, *Xanthomonadaceae*, *Rhodobacteraceae* and *Staphylococcaceae* were positively associated with frequent exacerbation and *Campylobacteraceae*, *Carnobacteriaceae*, *Corynebacteriaceae*, *Micrococcaceae*, *Neisseriaceae* and *Nocardiaceae* were positively associated with stability (Figure 5). *Pasteurellaceae*, *Streptococcaceae*, *Pseudomonadaceae* that were associated with stable patients (Figure 3), were not explanatory factors in this model (covariance between p1 and p2 was close to 0).

## Discussion

Microbial culture techniques have proven highly effective in identifying pathogens and managing acute infections. However, current sequencing approaches add doubts about the utility of these techniques in explaining the clinical paradigms in chronic polymicrobial infections [8]. Data on the polymicrobial communities in the lower airway of non CF Bronchiectasis using 16S rRNA gene amplicon sequencing is currently sparse. However, we identified, in common with previous studies, that in this NCFBr patient cohort, three taxa, *Streptococcaceae*, *Pseudomonadaceae* and *Pasturellaceae* were dominant (Additional file 2: Figure S1) [2,9,10]. We also showed that similar to CF bronchiectasis, the bacterial community was much more diverse than revealed by culture [2,11]. Contamination of the samples by oral flora is likely to occur during the production of the sputum. Although samples were washed [12] to minimise their impact, it is inevitable that oral bacteria are present in the samples and affect the bacterial communities found. The relationship between bacterial diversity, patient factors and disease progression in NCFBr remains to be determined. Rogers *et al*. [11] demonstrated a positive correlation between microbial diversity of the NCFBr lung with gender and lung function. In contrast, we and other studies [10] found no significant correlation between microbiome diversity and lung function, nor does our data support a significant difference in bacterial diversity between genders or gender significantly affecting the bacterial community structure in the NCFBr lung (Figure 1).

As previously reported [4] we found that 27% of the sputum samples tested were culture negative for recognised pathogens, although pyrosequencing demonstrated all had diverse bacterial communities. These included the anaerobic genera *Prevotellaceae*, *Streptococcaceae*, *Veillonellaceae* and *Actinomycetaceae* (Figure S1) that have been identified in other NCFBr microbial communities [9,11] as well as the bacterial communities found in CF and COPD lungs [13,14]. The role of these taxa remains to be elucidated, however, our analyses indicated that a number of them were correlated with exacerbations (Figure 3) or frequently exacerbating patients (Figure 5). A recent study has identified a relationship between neutrophilic airway inflammation and the total bacterial community suggesting a role for the whole lung microbiota in disease progression [15].

Our data indicates that the presence of culturable pathogens, particularly *P. aeruginosa* and *H. influenzae* are significant factors affecting bacterial communities in the NCFBr lung (Figure 1). This observation is relevant to the concept of core and satellite taxa in the chronically infected lung [16]. Core taxa are regarded as well adapted to the lung environment and able to persist, whereas satellite taxa are less well adapted and transient. If *P. aeruginosa*, *H. influenzae* and streptococci (Additional file 2: Figure S1) are core taxa, they may shape the community structure within a particular lung microbiome (Figure 1). For example, sputum samples from patients where *P. aeruginosa* had been persistently or intermittently cultured in the past contained significantly fewer taxa (44 versus 58, P = 0.012). This finding has previously been reported in CF studies where persistent colonisation was associated with mucoid and genetically adapted strains of *P. aeruginosa*[17]. There has been evidence to support the stratification of patients with NCFBr on the basis of *P. aeruginosa* culture with those chronically infected showing significantly lower lung function or poorer outcomes, including reduced bacterial diversity than those intermittently or never colonised patients [5-7,18,19]. Similarly, we found a significant reduction in FEV1% predicted (P < 0.001) between those patients persistently versus never colonised with *P. aeruginosa*. However, there was no significant link between low community diversity and FEV1% predicted.

As *Pseudomonas* was associated with a less diverse polymicrobial community we assessed its effect on the most prevalent pathogen in NCFBr. We observed that with culture and pyrosequencing data, *H. influenzae,* and *P. aeruginosa* were inversely related in sputum samples (Additional file 2: Figure S1). The pyrosequencing data showed when one is present (with one exception, patient 63), then the other did not contribute more than 1.5% to the total bacterial community profile (Additional file 2: Figure S1). In culture, *H. influenzae* was never co-isolated with *P. aeruginosa* (Table 1). This inverse relationship has been reported by others, for example, paediatric CF bronchiectasis patients showed a similar relationship between *P. aeruginosa* and *H. influenzae* in both culture and pyrosequencing analyses of microbial communities [10]. The implication is that both taxa cannot be regarded as part of a single ‘core’ microbiome. It remains unclear whether the inhibition of *H. influenzae* reflects antibiotic pressures, the arrival of *P. aeruginosa,* or a combination of these factors [19]. It could also reflect patient specific effects selecting for and leading to divergence in the airway communities. Such patient specific effects have been observed in other studies [20] but the underlying reasons are yet to be explained. We found *H. influenzae* was, however, present in patients with long term and repeated antibiotic therapy (data not shown). *P. aeruginosa* has been shown to inhibit the growth of *H. influenzae in vitro*[21] which suggests our observations may reflect competition between these two major pathogens in the human lung [22].

We modelled whether patients could be stratified on the basis of their microbiome, in particular, to determine whether patients undergoing a current exacerbation at sampling or those who were frequent exacerbators had a characteristic microbial community compared to stable patients or those who were infrequent exacerbators. Comparing acute exacerbations versus stable patients’ the bacterial community profiles indicated three groupings, a small exacerbating group, a group containing both stable and exacerbating patients and a third group of stable patients (Figure 2). We found particular taxa are correlated with different clinical states for example, 27 taxa including *Pasteurellaceae*, *Streptococcaceae*, *Xanthomonadaceae*, Burkholderiales, *Prevotellaceae* and *Veillonellaceae* were associated with acute exacerbations, whereas 11 taxa including *Pseudomonas* species correlated with stable clinical states (Figure 3). These observations, suggest that the bacterial community in the lung of exacerbating bronchiectasis patients has a more dynamic community composition than that seen in stable patients. It may be that the three groups identified based on community profiles are transient and individuals move in and out of them depending upon frequency of exacerbation, antibiotic treatment or other factors. Culture based studies of COPD suggest strain emergence is associated with exacerbations [23]. Although no patients were culture positive for *Burkholderia* spp., the presence of 1% of amplicons belonging to Burkholderiales, with one OTU accounting for 94% of the reads which was present, albeit in low numbers in 27% of the cohort, is notable as these organisms have not previously been considered pathogens in NCFBr.

We hypothesised that those individuals who frequently exacerbate would have significantly different bacterial community compositions and diversity compared to clinically stable patients. Soft-class modelling did not give a definitive answer, 39 profiles of both frequent exacerbators and stable patients were indistinguishable in the model, however, it did stratify a small group of 6 stable patient’s bacterial communities from those of a distinct group of 14 frequently exacerbating individuals (Figure 4). These profiles were not significantly different in terms of diversity or evenness (data not shown), but indicated certain taxa, particularly, *Rhodobacteraceae*, *Xanthomonadaceae Staphylococcaceae* and *Moraxellaceae* were associated with frequent exacerbations. The latter three taxa include established pathogens in acute exacerbations [24]. Here they are also implicated in increasing the frequency of exacerbation events. In contrast, the significance of taxa such as *Rhodobacteraceae* that are not routinely identified by standard culture is unknown. It is possible that they may be pathogenic, enhance the pathogenicity of clinically significant taxa or contribute to airway inflammation and decline in lung function [25,26].

In this study, there are inherent limitations; the patient cohort was consecutively recruited from an NCFBr out-patients clinic, hence, the administration of varying antibiotic regimens to individuals within the cohort may be a confounding factor. We identified 25 patients that had not received antibiotics for one month prior to sample collection. Ordination analyses (Figure 1) showed that these individuals did not have significantly different bacterial communities to those who were receiving antibiotic therapy. Our data suggests that antibiotics do not significantly perturb bacterial communities in the lower airway, however, transient impacts on abundance and diversity have been observed in longitudinal studies looking at microbial communities in sputum from CF patients [15,20]. The clinical benefit of antibiotic therapy in chronic lung infection may, therefore, be due to the reduction in bacterial load present [27]. A longitudinal study is required to confirm if a similar transient response is observed in NCFBr microbial communities. Other limitations are that in this cross sectional study we cannot gauge the level of temporal change within the lung microbiome, which if significant, may confound analyses showing differences in communities between individual patients. However, examining DGGE analyses of longitudinal samples from 35 individuals within this cohort (unpublished data) and other data using pyrosequencing approaches [10] shows that bacterial communities within an individual are relatively stable through time. A third issue, is that pyrosequencing relies on relatively short amplicons that lack sufficient resolution to confidently assign taxa to species, certainly not to strain-level. In many cases there is no independent culture data to support the metagenomic analyses and clinically significant strain differences are undetectable [24]. Finally, although exacerbations at time of sampling were clinically defined, and those in the preceding 12 months were determined where possible from patient records, some of the exacerbations episodes were self-reported by patients and as a result may not reflect the clinical definition used at time of sampling.

## Conclusions

In summary, we have demonstrated that the microbial community of the lower airway in NCFBr is dominated by three bacterial taxa *Pasteurellaceae*, *Streptococcaceae* and *Pseudomonadaceae*. We also show that the airway microbial community is much more diverse than indicated by culture and contains significant numbers of other genera including anaerobic *Prevotellaceae*, *Veillonellaceae* and *Actinomycetaceae*. We were unable to demonstrate a significant effect of antibiotic therapy, gender, or lung function on the diversity of the bacterial community. We did find presence of clinically significant culturable taxa; particularly *P. aeruginosa* and *H. influenzae* exerted a significant effect on the diversity of the bacterial community in the lung. Moreover, a high abundance of one of these pathogens is consistent with, but does not prove its causality in limiting the presence of the other taxa within the NCFBr lung bacterial community. This interaction requires further exploration. We also demonstrated that both acute exacerbations, the frequency of exacerbation and episodes of clinical stability cause, in some patients, a significantly different bacterial community structure, that are associated with a presence of particular taxa in the NCFBr lung.

## Methods

### Ethics statement

Ethical approval for the study was by the National Research Ethics committee (ref 12/NE/0248). Participants provided written informed consent prior to entry in the study.

### Patient cohort

The inclusion criteria were adult out-patients attending a specialist bronchiectasis clinic in North East (NE) England, U.K. with a clinical diagnosis of NCFBr confirmed by High Resolution CT scanning. All non-CF aetiologies were included with idiopathic and post infectious aetiologies predominant; a minority were immunodeficiency related, rheumatoid arthritis or COPD related (Additional file 1: Table S1). Exclusion criteria were radiological evidence of bronchiectasis without sputum production or entry into any other clinical trial. Aetiological designation was based upon a published protocol [2]. Cystic fibrosis genotyping and/or sweat testing was undertaken as per national guidelines [28]. Recruitment was on an unselected consecutive basis. Information on bronchiectasis aetiology, patient gender, age, 12 month previous history of exacerbations, forced expiratory volume in one second (FEV1), and maintenance chronic antibiotic therapy (Azithromycin 250 mg once daily, thrice weekly) or inhaled antibiotic therapy was collected by reviewing patient case notes (Additional file 1: Table S1). For current clinical status at time of sampling an exacerbation was defined as the presence of increased cough, malaise with increased sputum volume and purulence requiring antibiotic treatment. Frequent exacerbators were defined as those patients who reported more than 3 episodes over the preceding 12 months [28]. 25 patients recruited were found to have received neither antibiotics for acute treatment of an exacerbation or azithromycin for one month prior to sampling. Patients were classed as current exacerbators if they reported an increase beyond their baseline level of symptoms that were consistent with an exacerbation as defined by national Bronchiectasis guidelines [28]. Lung function was determined by forced expiratory volume in one second (FEV1% predicted) in all patients according to national standards.

### Collection of sputum samples and microbial culture

Spontaneously expectorated sputum samples were collected from consecutive outpatients within a cohort of adult NCFBr patients. The samples were washed with phosphate-buffered saline to remove any contamination from oral flora [12]. Each sample was homogenised with Sputasol (Oxoid) and divided into two aliquots, one for subsequent DNA extraction and one for immediate culture, performed in accordance with national standard methods in an accredited UK clinical laboratory. Briefly, 10 μL aliquots of homogenised sputum were cultured onto Columbia blood agar and Chocolate agar plus bacitracin. The sample was subsequently diluted 1/100 in sterile saline (0.85%) and 10 μL of this was cultured onto chocolate agar and incubated in air plus 5% carbon dioxide (37°C, 48 h). Isolates were identified by matrix assisted laser desorption ionisation time-of-flight (MALDI-TOF) mass spectrometry (Bruker Daltonics) and, where necessary, appropriate API kits (bioMérieux) [29]. Information, from up to 10 years previously on prior *P. aeruginosa* status, was collected (Additional file 1: Table S1). Persistent infection was defined as isolation ofa taxa from previous sputum samples with a minimum requirement of having been cultured on two or more occasions [2] based upon current and prior sputa culture data. Intermittent colonisation was defined as isolation of taxa from a patient’s sputa preceded or followed by sputa that was culture negative. DNA was extracted from 0.5 ml of each sputum sample using the MoBio Ultraclean Microbial DNA isolation kit (MoBio, CA, USA) according to the manufacturer’s protocol. A negative control where template DNA was replaced with sterile distilled water was prepared with the same reagents. Extracted DNA was quantified with a NanoDrop 1000 Spectrophotometer (Thermo Scientific).

### 454 Pyrosequencing

From standardised concentrations of template DNA a portion of 16S rRNA gene (position 341 to 907; *Escherichia coli* numbering) was amplified using the primer set 341 F and 907R [30]. DNA sequencing was performed using the 454 GS FLX Titanium Sequencing System (Roche, IN, USA) by the Research and Testing Laboratory (RTL, TX, USA) using previously described methods [31].

The raw sequencing reads were quality filtered in QIIME 1.6.0 [32] using the split-library.py script. Remaining high quality sequences were clustered into operational taxonomic units (OTUs) at 97% similarity using UCLUST [33]. Representative sequences for each OTU were aligned using PyNAST [34] and taxonomic identities were assigned using RDP-classifier (version 2.2) [35] with 50% as confidence value threshold. Detection of potentially chimeric sequences was performed using ChimeraSlayer [36] and chimeric sequences were removed from downstream analysis prior to tree building using FastTree [37]. Analyses were run to the family level of taxonomic resolution and also to species level (Additional file 3: Table S2). However, species level identification can only be regarded as putative given the relatively short fragment of the 16S rRNA gene sequenced. Sequences were deposited in MG-RAST under the accession numbers 4534396.3-4534463.3.

### Polymicrobial community and statistical analyses

Clinical parameters were tested using Students t-tests and probability (P) values <0.05 deemed to be statistically significant. Distribution of data was tested using Shapiro-Wilk test (α =0.05). Community sequence data were first analysed by de-trended correspondence analysis (DCA). The DCA axis was >3.5 indicating that canonical correspondence analysis (CCA) was the most appropriate ordination method). Direct ordination was performed with Monte Carlo permutation testing (499 permutations) using CANOCO 4.5 [8]. Constrained (canonical) analyses show variation between the sample profiles that can be explained by the measured categorical and continuous variables of interest e.g. FEV1% predicted or gender (Table 1).

Subsequently, processed sequencing matrices were analysed using soft class modelling (PLS-DA) to investigate trends in community composition and identify those taxa from the 454 analyses that contribute most to community variation.

### Soft-Class modelling of pyrosequence data

Patient samples were classified according to two main parameters; the first, current clinical status at time of sampling (exacerbating versus stable) and secondly, overall 12 month exacerbation history (frequent exacerbators; >3 events per annum (M1) versus infrequent exacerbators ≤3 event per annum (M2)). Assessment of overall community composition and relationship between clinically important pathogens namely *Pseudomonadaceae* (including *Pseudomonas aeruginosa*), *Pasteurellaceae* (including *Haemophilus influenzae*), *Streptococcaceae* (including *Streptococcus pneumoniae*), *Enterobacteriaceae*, (including *Escherichia coli*, *Serratia liquefaciens and Morganella morganii*), *Xanthomonadaceae* (including *Stenotrophomonas maltophilia*) and members of the genera *Veillonella*, *Prevotella*, and *Neisseria* were explored. Data were analysed using supervised discriminant analysis to explore the linear regression between the microbial community structures (*X*) and the defined descriptive variables (*Y*). Sputum from patients reporting clinical stability at time of sampling were used as matched controls against samples taken from exacerbating patients.

Group classification was based on within patient sampling through time, exacerbation frequency (>3 exacerbation events per annum), current clinical status (stable versus exacerbated) and presence of major pathogens to assess the effects of these parameters on microbial community assemblage (SIMCA, Umetrics). To check that data was adhering to multivariate normalities, Hotelling’s *T*^*2*^ tolerance limits were calculated and set at 0.95. Outliers that were deemed to be moderate, and unable to shift in the model plane were also subjected to a DModX (Distance to model in the X space) calculation to assess those outliers that did not fit the model well, and deviate from the normal F-distribution (critical distance 0.05).

## Competing interests

The authors declare that they have no competing interests.

## Authors’ contributions

PP carried out the collection of the pyrosequencing and patient data, contributed to the statistical analyses of these data sets and helped draft the manuscript. HJ coordinated the collection of the patient specific data and helped to draft the manuscript. AP undertook the culture based analyses of samples. JDP participated in the study design, culture based analyses and coordination and helped to draft the manuscript. CJS generated sequence information and contributed to the statistical analysis. AN contributed to the statistical analyses of these data sets and helped draft the manuscript. CL participated in the design of the study and performed the statistical analysis. DLS participated in the generation and analysis the sequence data. SPC conceived of the study, and participated in its design and coordination and drafted the manuscript. ADS conceived of the study, and participated in its design and coordination and helped to draft the manuscript. All authors read and approved the final manuscript.

## Supplementary Material

Additional file 1: Table S1 Clinical information on patient cohort. Click here for file

Additional file 2: Figure S2 Family level bar plot of all samples that underwent 454 pyrosequencing. Click here for file

Additional file 3: Table S2 Analyses of pyrosequence data to species level giving total number of reads, putative identification of each taxon and their contribution expressed as percentage of total reads. Click here for file
