# Supplementary figures and images for: Polymicrobial airway bacterial communities in adult bronchiectasis patients
Source: BMC Microbiol. 2014 May 20;14:130. doi: 10.1186/1471-2180-14-130 (PMC4031157; doi:10.1186/1471-2180-14-130)

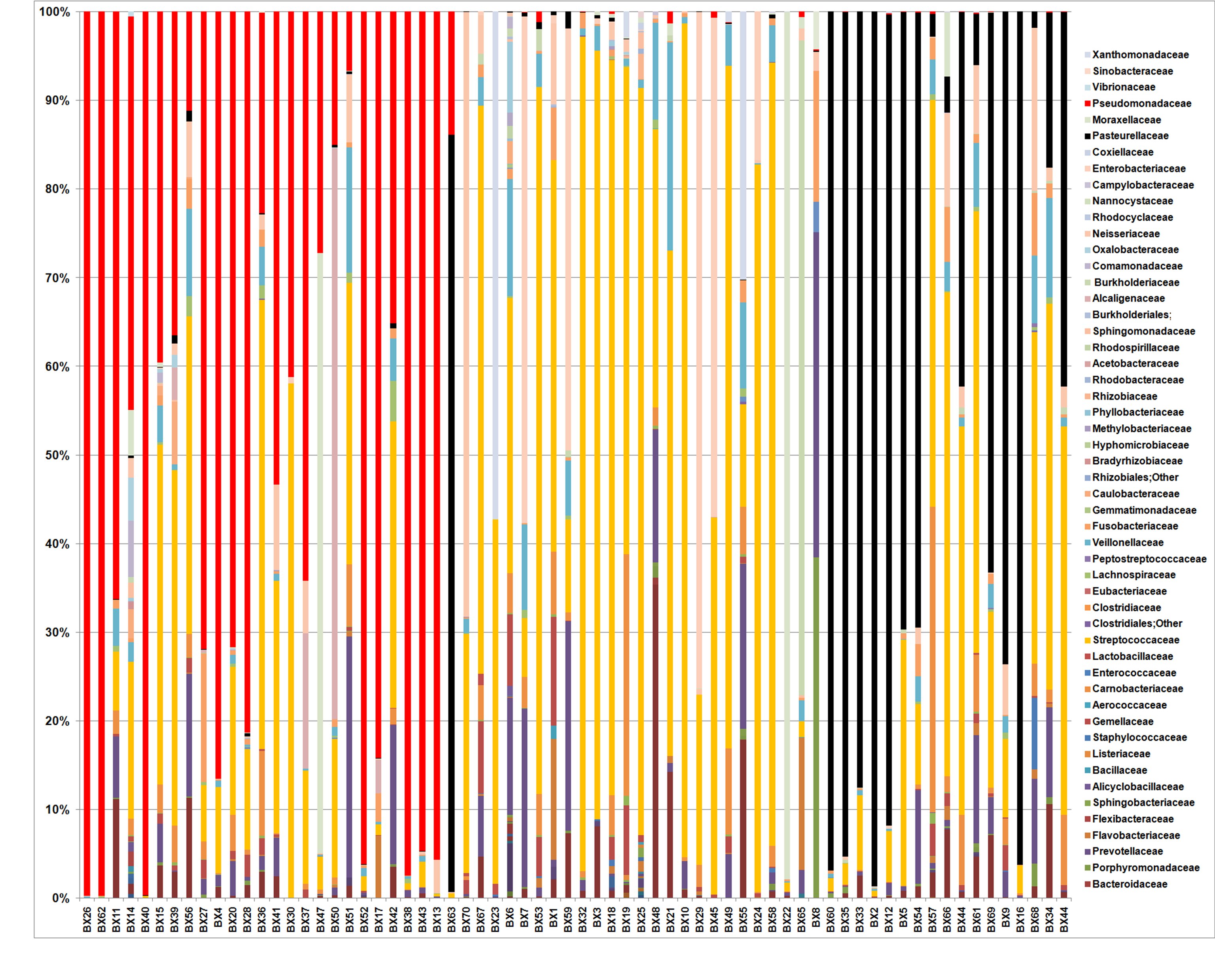

Supplement: Additional file 2: Figure S2 — Family level bar plot of all samples that underwent 454 pyrosequencing. [file 1471-2180-14-130-S2.tiff]
